# First study on capsular serotypes and virulence factors of *Pasteurella multocida* isolates from Phan Rang sheep in Vietnam

**DOI:** 10.14202/vetworld.2023.281-290

**Published:** 2023-02-14

**Authors:** Phu Van Nguyen, Cong Tuan Le, Xuan Huy Nguyen, Tuan Manh Nguyen, Kim Cuc Thi Nguyen

**Affiliations:** 1 Institute of Biotechnology, Hue University, Road 10, Phu Thuong, Thua Thien Hue, Vietnam; 2 Department of Environmental Science, University of Sciences, Hue University, Thua Thien Hue, Vietnam; 3 Department of Science, Technology and International Relations, Hue University, Thua Thien Hue, Vietnam; 4 Institute of Life Science, Thai Nguyen University of Agriculture and Forestry, Quyet Thang, Thai Nguyen, Vietnam

**Keywords:** antimicrobial resistance, biofilm formation, capsular type, Pasteurella multocida, Phan Rang sheep

## Abstract

**Background and Aim:**

*Pasteurella multocida* is considered as a main factor mediating pneumonic pasteurellosis in ruminants, including sheep. It is also a current threat to Phan Rang sheep in Vietnam. This study aimed to characterize *P. multocida* isolated from Phan Rang sheep, their antibiotic resistance profile, and the prevalence of some virulence-associated genes of these strains.

**Materials and Methods:**

Bacteria were isolated on brain heart infusion, 10% sheep blood agar plates, and screened by biochemical tests. The polymerase chain reaction technique was used with specific primers to identify *P. multocida*, the presence of virulence-associated genes, and serotypes of isolates. Antimicrobial susceptibility and biofilm formation of isolates were examined using the disk diffusion method and crystal violet-based method, respectively.

**Results:**

A total of 41 *P. multocida* strains were isolated from 485 samples from clinically sick and healthy sheep. Of the isolates, 58.53% were serotype A, 9.75% were serotype B, and 31.71% were serotype D. Healthy animals were infected with serotype D only. All 15 virulence genes were identified in all strains isolated from clinically sick sheep, while strains isolated from healthy sheep carried 11/15 virulence genes tested. Among virulence-associated genes *exbB, exbD*, *tonB*, *ompA*, *oma87*, *fimA, hgbA*, and *nanB* were detected in over 90% of isolates, whereas *hgbB, nanH, tbpA* and *pfhA* were less frequent. Interestingly, *pmHAS* and *tadD* were highly prevalent in capsular type A strains, whereas the *toxA* gene was detected in capsular type D strains only. All of the isolated strains were fully susceptible to enrofloxacin, ciprofloxacin, neomycin, and ofloxacin. About 92.68% were susceptible to chloramphenicol and 90.24% to amikacin, but there was high resistance to erythromycin, tetracycline, and amoxicillin. Our results reveal that 53.65% of 41 isolates could produce biofilm, whereas 46.34% could not.

**Conclusion:**

*Pasteurella multocida* from Phan Rang sheep possess many virulence genes and resistance to several common antibiotics such as erythromycin, tetracycline, and amoxicillin. The results are an important warning regarding antibiotic resistance of *P. multocida*.

## Introduction

Vietnam has thermotolerant sheep that have been reared in Ninh Thuan province for over a century, named Phan Rang sheep [1]. The sheep are well adapted to tropical weather, although their genetic origin has not been identified yet. The sheep were also experimentally reared in different regions of Vietnam other than Ninh Thuan and were considered a local candidate for adapting to global warming. However, research on this breed is still limited, especially in disease-related data. *Pasteurella multocida* is an important pathogen that causes pasteurellosis, one of the most frequent and dangerous diseases in sheep rearing worldwide [2–4]. In Vietnam, *P. multocida*, from avians and pigs, has been isolated and characterized [5–7], but no related information has been reported in ruminants, particularly in Phan Rang sheep.

*Pasteurella multocida*, in normal conditions, can live quietly in the respiratory tract of healthy animals without causing disease-related symptoms [8]. However, the bacteria become threatening in stress conditions, such as extreme weather, water shortages, or cramped barns, which are proposed to compromise the immune system of animals [9]. At least 5 serotypes of *P. multocida* have been reported based on cellar capsules, including A, B, C, D, E, and F [10, 11]. In sheep, serotype A, particularly sub-serotype A1 and A3, is more predominant than D or B serotypes [11, 12]. Besides cellular capsules, some virulence factors required for *in vivo* reproduction and disease development (lipopolysaccharide) or cellular survival (iron-regulated or iron-acquisition proteins) have been identified in *P. multocida* as well as other virulence-associated factors such as toxins, fimbriae, adhesins, sialic acid metabolism, hyaluronidase, or outer membrane proteins [13–15].

Antimicrobial agents are widely used to control and cure pasteurellosis disease in poultry and livestock [16]. However, their use has to be carefully considered since correct dosage and usage are commonly not followed, particularly in developing countries leading to the onset of antibiotic resistance, a global problem in animal husbandry and human health [17–19]. To date, antibiotic-resistant *P. multocida* has been reported in different food-producing animals, in particular, a high prevalence of antibiotic resistance was observed in pigs [5, 18], in chickens [20], and small ruminants like sheep [21, 22]. Hence, host origin may be a critical factor in the evolution of antibiotic resistance in *P. multocida*.

Therefore, this study aimed to investigate, for the first time, serotypes of *P. multocida* isolated from Phan Rang sheep and their antibiotic resistance profile, as well as to estimate the prevalence of some virulence factors in these strains.

## Materials and Methods

### Ethical approval

Ethical approval from the Institute of Biotechnology, Hue University, to conduct this study was not required. However, the sample collection was conducted as per the standard sample collection procedure without any harm to animals.

### Study period and location

The study was conducted from April 2021 to October 2022 at the Institute of Biotechnology, Hue University, Vietnam.

### Sample collection and bacterial isolation

Samples were collected from Bac Ai (11°42’06.2”N 108°54’38.0”E), Ninh Phuoc (11°30’09.1”N 108°52’02.1”E), Ninh Hai (11°36’09.6”N 109°07’36.9”E), and Thuan Bac (11°40’53.5”N 109°05’13.8”E) of Ninh Thuan, Province, Vietnam. This province is the site of the greatest number of live sheep and the greatest sheep production in Vietnam. A total of 485 Nasal swab samples were collected from 185 healthy sheep and 300 clinically sick sheep (coughing, breathing discomfort, and nasal discharge). The swab sample was transported ina cool box (4°C) to the Laboratory of the Cell, Institute of Biotechnology, Hue University for further processing.

For preliminary isolation, all samples were plated on brain heart infusion (BHI) (HiMedia, India), 10% sheep blood agar plates and incubated at 37°C for 48 h. After that, bacteria cells from suspected colonies were examined by testing their ability to grow on Macconkey agar (HiMedia), gram’s staining, and microscopy at 100× magnification. The strains that were unable to grow on MacConkey agar and that were Gram-negative were further characterized by biochemical tests using Microgen™ GnA + B-ID System (Microgen, UK) to identify *P. multocida*.

### Polymerase chain reaction (PCR) detection of *P. multocida*

Cells from a single colony on a blood agar plate were inoculated in BHI broth at 37°C for 12 h in a shaking incubator. Bacterial culture was then collected by centrifugation at 18000× *g* for 1 min. Genomic DNA was isolated using the DNA isolation kit (Qiagen, Germany) according to the manufacturer’s instructions. A specific PCR assay was carried out to confirm the presumptive isolates of *P. multocida* by amplifying a fragment of the kmt1 gene as described by Townsend *et al*. [23] using specific primers as shown in Table-1 [23–25]. The PCR reaction was performed in PCR thermal cyclers (PTC200, MJ, USA) using PCR standard master mix (Canvax Biotech, Spain) in a total volume of 25 μL containing 0.5 µL of each primer (10 pM) and 1 μL of template DNA. The PCR conditions consisted of an initial denaturation at 95°C for 5 min, followed by 35 cycles of denaturation at 95°C for 1 min, 1 min for primer annealing at 52°C extension at 68°C for 1 min, and a final extension at 68°C of 5 min. About 5 μL of PCR products were electrophoresed on a 1.5% agarose gel with safe red (Intron, Korea) for visualization under the MUPID^®^ One LED Illuminator (Nippon genetics, EU). DNA of P *. multocida* strains was used as a positive control, while PCR water was used as a negative control, as published by Vu-Khac *et al*. [5].

**Table-1:** Primers used for the identification of *P. multocida* isolates and for the detection of capsular types and virulence-associated genes in *P. multocida.*

Target gene	Description	Sequence (5′ – 3′)	Annealing temp (°C)	size (bp)	References
*kmt1*	Detection of all *P. multocida*	CCTCCGACTAACACCCAAGT TGGGCTTGTCGGTAGTCTTT	56	633	This study
*hyaD-hyaC*	Serogroup A *cap* gene	TGCCAAAATCGCAGTGAG TTGCCATCATTGTCAGTG	50	1044	[24]
*bcbD*	Serogroup B *cap* gene	CATTTATCCAAGCTCCACC GCCCGAGAGTTTCAATCC	51	760	[24]
*dcbF*	Serogroup D *cap* gene	TTACAAAAGAAAGACTAGGAGCCC CATCTACCCACTCAACCATATCAG	54	657	[24]
*ecbJ*	Serogroup E *cap* gene	TCCGCAGAAAATTATTGACTC GCTTGCTGCTTGATTTTGTC	50	511	[24]
*fcbD*	Serogroup F *cap* gene	AATCGGAGAACGCAGAAATCAG TTCCGCCGTCAATTACTCTG	54	851	[24]
*ompA*	Outer membrane protein A	CGCATAGCACTCAAGTTTCTCC CATAAACAGATTGACCGAAACG	55	201	[23]
*oma87*	Outer membrane protein 87	GGCAGCGAGCAACAGATAACG TGTTCGTCAAATGTCGGGTGA	55	838	[23]
*fimA*	Fimbriae	CCATCGGATCTAAACGACCTA AGTATTAGTTCCTGCGGGTG	55	866	[23]
*pfhA*	Filamentous hemagglutinin	TTCAGAGGGATCAATCTTCG AACTCCAGT TGGTTTGTCG	55	286	[23]
*nanB*	Neuraminidases	CATTGCACCTAACACCTCT GGACACTGATTGCCCTGAA	55	555	[23]
*nanH*	Neuraminidases	GTGGGAACGGGAATTGTGA ACATGCCAAGTTTGCCCTA	55	287	[23]
*exbB*	Iron regulated and acquisition factors	TTGGCTTGTGATTGAACGC TGCAGGAATGGCGACTAAA	55	283	[23]
*exbD*	Iron regulated and acquisition factors	CGTTCTGATTACAGCCTCTT AACGAAATCTTGGAAACTGG	55	247	[23]
*tonB*	Iron acquisition related factors	CGACGGTGAAACCTGAGCCA CCGAGCGATAAGCATTGACT	55	261	[23]
*tbpA*	Iron acquisition related factor	TGGTTGGAAACGGTAAAGC TAACGTGTACGGAAAAGCC	54	728	[23]
*hgbA*	Hemoglobin binding protein	TGGCGGATAGTCATCAAG CCAAAGAACCACTACCCA	51	419	[25]
*hgbB*	Hemoglobin binding protein	ACCGCGTTGGAATTATGATTG CATTGAGTACGGCTTGACAT	55	788	[25]
*pmHAS*	Hyaluronidase	TCAATGTTTGCGATAGTCCGTTAG TGGCGAATGATCGGTGATAGA	51	430	[23]
*toxA*	Dermonecrotic toxin	CTTAGATGAGCGACAAGG GAATGCCACACCTCTATAG	55	864	[23]
*tadD*	Adhesin	TCTACCCATTCTCAGCAAGGC ATCATTTCGGGCATTCACC	55	416	[23]

*P. multocida=Pasteurella multocida*

### Capsular typing and virulence-associated gene detection

The capsular types of *P. multocida* isolates were determined by a multiplex PCR using specific primers targeting capsule biosynthesis genes (cap A, B, D, E, and F) as described previously [24].

The detection of virulence-associated genes was investigated by PCR with specific primers. The details of all primer pairs and annealing temperature are listed in Table-1 [23–25]. The PCR conditions were carried out as previously described [24, 25].

### Antibiotic susceptibility

An antimicrobial susceptibility test was carried Polymerase chain reaction

out by the disk diffusion method in accordance with the guidelines of Clinical and Laboratory Standards Institute (CLSI) [26]. In the pipeline for testing, all *P. multocida* isolates were grown in BHI broth until OD_600_ reached 0.5 and then were spread over Mueller-Hinton plates (HiMedia). After a few minutes of surface drying, 12 antibiotic discs (Mast group, England) with concentrations as follows: Amikacin (30 μg), amoxicillin (10 μg), ampicillin (10 μg), ciprofloxacin (5 μg), chloramphenicol (30 μg), erythromycin (15 μg), gentamicin (10 μg), kanamycin (30 μg), neomycin (10 μg), ofloxacin (5 μg), enrofloxacin (5 μg), and tetracycline (30 μg) were placed equally-spaced on the surface of the plates and incubated at 37°C for 24 h. The antibiotic susceptibility of isolates was based on the inhibition zone diameter standards provided by the CLSI [26]. *Escherichia coli* ATCC 35218 and *Staphylococcus aureus* ATCC 25923 were used as the quality control strains.

### Biofilm formation assay

The biofilm formation capacity of *P. multocida* isolates was examined according to Sager *et al*. [27] with some modifications. Briefly, bacterial cells of *P. multocida* isolates were grown in BHI broth overnight at 37°C. Cultures were harvested by centrifugation at 14000× *g* for 5 min; cells were then resuspended in tryptic soy broth without glucose (Merck, Germany), and were adjusted to OD_600_ = 1 (equal to approximately 3 × 10^8^ CFU/mL). Subsequently, 150 μL of the diluted culture was inoculated in triplicate to 96-well plates (SPL Life Science, Korea) and incubated at 37°C in static conditions. After 24 h of incubation, non-adhesive cells and culture medium were removed, subsequently, adhered cells of biofilms in the well were washed 3 times with distilled water and stained by 150 μL of 1% crystal violet (Merck) for 15 min. After that, the plates were rinsed 3 times, before adding 150 μL of 70% ethanol and incubated for 15 min. Finally, 100 μL of soluble crystal violet was transferred to a new 96-well plate, and absorbance was determined by optical density (OD) at wavelength 540 nm. The experiments were carried out with three replicates. The biofilm formation ability of isolates was calculated according to the method described by Stepanović *et al*. [28]. In brief, the strain is classed as a non-biofilm producer when the average OD is less than the control value (ODc), weak biofilm producer means ODc < OD ≤ 2 × ODc, moderate biofilm producer as the following formation: 2 × ODc < OD ≤ 4 × ODc, and strong biofilm producer when OD > 4 × ODc. In all experiments, ODc (control measurement) was carried out in a microtiter plate without cells.

## Results and Discussion

### *Pasteurella multocida* isolation and capsular detection

**Figure-1:**
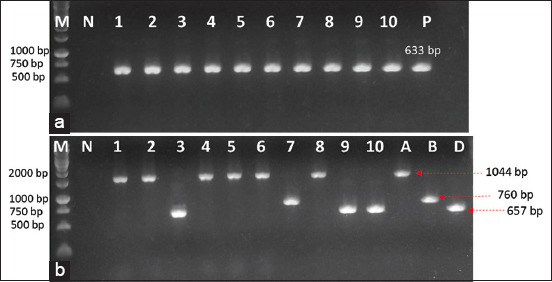
*Pasteurella multocida*-specific polymerase chain reaction (PCR) assay by *kmt1* primers and multiplex PCR for capsular typing of *P. multocida* isolates. (a) Verification of *P. multocida* by PCR. Lane M, 1 kb DNA ladder marker (ThermoScientific, USA); lanes 1–10: detection of *P. multocida* from isolates (1–10); lane P: Positive control (633 bp), lane N: Negative control. (b) Capsular identification in isolated *P. multocida* by multiplex PCR assay. Lane M, 1 kb DNA ladder marker (ThermoScientific, USA); lane N, negative control; lanes 1–10 isolates (1–10); A positive control of capA (1044 bp); B, positive control of capB (760 bp); and D, positive control of capD (657 bp).

The suspected *P. multocida* isolates were obtained from blood agar and biochemical tests, with typical characteristics bacterial cells were small, Gram-negative and coccobacillus with bipolar staining features; the colonies were gray, viscous with odor and non-hemolytic. They were further verified by PCR. Specific PCR assay using Gram-negative KMT1 primers identified 41 isolates as *P. multocida* (The representative ten isolates of *P. multocida* are shown in Figure-1) with an incidence of 8.45% (41/485 samples), of which 38 isolates were successfully isolated from clinically sick sheep (38/300) and three isolates from healthy sheep (3/185). This average percentage result is higher than that of Fernandez *et al*. [13], in which the incidence was 6.2% (37/598 *sample*s), but lower than the reports of Shayegh *et al*. [29], which was 9.07% (47/518 *sample*s). Our results also indicated that *P. multocida* was significantly higher in clinically sick sheep (12.66%) compared to healthy ones (1.62%). These results are consistent with a previous study, in which 18.62% *P. multocida* was isolated from clinically sick lambs compared to healthy ones 0.49% [13]. However, there was not much difference from the study of Shayegh *et al*. [29], in which prevalence was 8.14% and 9.27% for healthy sheep and diseased sheep, respectively. The difference observed here may be from the size of the sample investigation and the characteristics of the local animal studied.

Based on PCR serotyping of 41 *P. multocida* isolates, the *capA, capB*, and *capD* genes were detected in 24 *(*58.53%), 4 *(*9.75%), and 13 *(*31.71%) isolates, respectively. However, the *capE*, and *capF* genes were not detected in all of the isolates (Table-2 and Figure-1). A compatible result was also observed in a study conducted by Aski and Tabatabaei [30], in which the prevalence of *capA*, *capB*, and *capD* genes in *P. multocida* isolates from sheep was 45.45, 13.63, and 34.09%, respectively. However, in some cases, the *capB* gene was absent in *P. multocida* isolates from sheep [13, 15]. Nevertheless, data in the literature suggest that capsular types A and D are common among *P. multocida* strains isolated from sheep and goats [13, 15, 29, 30].

**Table-2:** The prevalence of virulence genes associated with capsule serotypes among 41 isolates of *P. multocida.*

*P. multocida* isolation	Capsular type	Number of isolates (%)	Virulence gene (%)														

			*fimA*	*pfhA*	*pmHAS*	*toxA*	*hgbA*	*hgbB*	*tbpA*	*exbB*	*exbD*	*tonB*	*nanB*	*nanH*	*tadD*	*ompA*	*oma87*
Healthy (185)	A	0 (0)	-	-	-	-	-	-	-	-	-	-	-	-	-	-	-
	B	0 (0)	-	-	-	-	-	-	-	-	-	-	-	-	-	-	-
	D	3 (7.32)	3 (7.32)	0 (0)	0 (0)	3 (7.32)	3 (7.32)	0 (0)	1 (2.44)	3 (7.32)	3 (7.32)	3 (7.32)	2 (4.88)	0 (0))	1 (2.44)	3 (7.32)	3 (7.32)
Sick (300)	A	24 (58.53)	22 (53.65)	6 (14.63)	24 (58.53)	0 (0)	23 (56.09)	17 (41.46)	20 (48.78)	24 (58.53)	24 (58.53)	24 (58.53)	23 (56.09)	14 (34.14)	17 (41.46)	24 (58.53)	24 (58.53)
	B	4 (9.75)	3 (7.32)	1 (2.44)	1 (2.44)	0 (0)	3 (7.32)	2 (4.88)	3 (7.32)	4 (9.75)	4 (9.75)	4 (9.75)	2 (4.88)	3 (7.32)	2 (4.88)	4 (9.75)	4 (9.75)
	D	10 (24.39)	10 (24.39)	1 (2.44)	0 (0)	10 (24.39)	10 (24.39)	6 (14.63)	9 (21.95)	10 (24.39)	10 (24.39)	10 (24.39)	10 (24.39)	7 (17.07)	1 (2.44)	10 (24.39)	10 (24.39)
Total (485)			38 (92.68)	8 (19.51)	25 (60.97)	13 (31.71)	39 (95.12)	25 (60.97)	33 (80.48)	41 (100)	41 (100)	41 (100)	37 (90.24)	24 (58.53)	21 (51.22)	41 (100)	41 (100)

*P. multocida=Pasteurella multocida*

Interestingly, only three *P. multocida* strains were isolated from healthy sheep and all of them (3/3) were detected as capsular type D. On the other hand, among 38 *P. multocida* strains that were isolated from clinically sick sheep, serotype D made up 26.31% (10/38). These findings are in line with the previous study [13], but completely different from those reported by Shayegh *et al*. [29], which indicated that capsular type D was isolated only from diseased sheep, whereas capsular type A presented in both diseased and healthy sheep. Similarly, Khamesipour *et al*. [19] reported that capsular type A was detected in both pneumonic lung and healthy lung isolates. In agreement with the literature, no strong correlation between serotype and the prevalence of *P. multocida* was identified. The distribution and prevalence of *P. multocida* serotypes have been reported in other studies and vary from region to region, host to host. Serotype D was found most commonly in cattle (82.6%) [31], and varied in sheep (6.3 to 57.1%) [13, 29]. Meanwhile, serotype A was predominantly found in sheep (83.3%) [29], and swine (about 50%) [5, 32]. In addition, serotype B was absent in a previous study [13, 29], but it was detected in the present study. Similarly, *P. multocida* type B was also detected in cattle in Vietnam [33].

### Detection of virulence-associated genes

**Figure-2:**
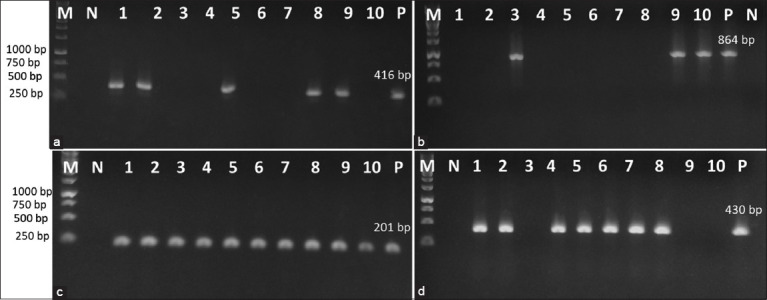
Polymerase chain reaction assay checking the prevalence of virulence genes of *Pasteurella multocida* isolated from Phan Rang sheep. Lane M, 1 kb DNA ladder marker (ThermoScientific, USA); lanes 1–10: Samples, lane N: Negative control, lane P: Positive control. (a) Detection of *toxA* gene; (b) detection of *tadD* gene; (c) detection of *ompA* gene; (d) detection of *pmHAS* gene.

The frequency of 15 virulence genes of *P. multocida* isolates was detected by PCR (Table-2) and the representative gel pictures for the distribution of four virulence genes in 10 isolates are shown in Figure-2. The results showed that all isolates carried iron-acquisition encoding genes (*exbB, exbD* and *tonB*), and outer membrane protein genes (*ompA, oma87*). The *fimA* gene encoding an adhesin was detected in 92.68% of isolates, whereas another gene in the same functional group (*pfhA*) was detected as low as 19.51%. The incidence of *toxA, tadD*, *pmHAS*, and *tbpA* genes was 31.71, 51.22, 60.97, and 80.48% in all the isolates, respectively. Among the heme receptors (*hgbA* and *hgbB*) studied, *hgbA* was detected in 95.12% of isolates, particularly in all type D isolates, while 60.97% of isolates carried the *hgbB* gene. Similarly, *nanB* was present in 90.24% of isolates, compared to a lower frequency of *nanH* (58.53%).

It is known that *P. multocida* has a number of virulence factor encoding genes that are essential for bacterial infection [34]. Among these genes, *fimA* and *pfhA* encode factors that mediate bacterial cell adherence to host cells, one of the first steps of bacterial infection [21]. The previous studies reported that the *pfhA* gene has a very high prevalence and irrespective of its capsule type [21, 31, 35]. However, in this study, we observed a relatively low occurrence of the *pfhA* gene in isolates from sheep. These results are consistent with other studies [25, 29, 30] that reported the low distribution of this gene with great variance among the strains of *P. multocida*. Another factor gene *fimA* which has not been well characterized, exhibited high prevalence in this study; similar results were also observed in the studies of Aski *et al*. [30], Tang *et al*. [18] and Ewers *et al*. [25], shown that more than 80% isolates harbored this gene. These findings suggest that the *fimA* gene may play an important role in bacterial adhesion.

Meanwhile, the *tadD* gene encoding a non-specific adhesion factor was identified here in more than 50% of isolates, of which 70.8% (17/24 isolates) of capsular A carry the *tadD* gene, whereas 15.3% (2/13) isolates of capsular D carry the *tadD* gene (Figure-2a). Comparison of the findings with those of the previous studies supports the suggestion that the distribution of *tadD* in capsular A is more frequent than it is in capsular D [18, 25, 30, 36]. Other adhesion-related genes, *nanB* and *nanH* (sialidases) play a significant role in the colonization of epithelial surfaces [37]. In agreement with the previous studies [25, 30], the *nanB* gene was present in most isolates (>90%), whereas the *nanH* gene was detected in <60% of isolates. Conversely, *nanH* was detected in 100% of Indian bovine isolates, while *nanB* was absent in the study of Verma *et al*. [31]. These again suggest that the varying prevalence of *nanH* depends on the host or host origins and geographical location as previous studies [21, 25].

Iron is an essential nutrient for nearly all bacterial pathogens [38], *P. multocida* may turn on the expression of *hgbA*, *hgbB*, and *tbpA* genes, and TonB complex genes (*exbB, exbD*, and *tonB*) for acquiring iron from different heme iron sources [39]. In this study, the prevalence of three genes in the TonB complex was 100% in all isolates. These results are consistent with the previous studies [18, 25] and reasonable since iron uptake depends on the energy generated from the TonB complex [34, 39]. In contrast, the regular distribution of two genes (*hgbA* and *hgbB*) mediating iron-acquisition directly from the heme component was very different. About 95.12% of isolates carried the *hgbA* gene, whereas *hgbB* was present in 60.97% of isolates. These findings broadly supported those of other studies [21, 25, 30] in which the *hgbA* gene is found to be highly prevalent (>95%) among *P. multocida* isolates. In contrast, *hgbB* gene distribution varied and depended on strains of different host origins. Notably, Shayegh *et al*. [29] reported that the regular distribution of the *hgbB* gene is 36.36% among sheep isolates, our study revealed a higher prevalence (60.97%) of this gene.

The TbpA protein has been described as an important virulence factor, and the *tbpA* gene is present in small ruminant strains [21, 25, 35, 40]. Our results revealed that a high frequency (80.48%) of the *tbpA* gene was detected among isolates from sheep; similar results were found in the studies of Katsuda *et al*. [36] and Sarangi *et al*. [21]. However, the frequency was lower compared to the study of Fernandez *et al*. [13], who found that the *tbpA* gene was present in all *P. multocida* strains from sheep.

Interestingly, *pmHAS* and *toxA* exhibited distinctive relations to serotypes (Figures-2b-d); the *pmHAS* was detected in all the capsular type A isolates with a prevalence of 58.53% (24/41 isolates), whereas the *toxA* gene was correlated to capsular type D only with 31.71% of the distribution of 13/41 isolates. Similar results were also observed, in which several virulence genes, including *pmHAS* and *toxA* have been reported as factors linked to specific capsular types [13, 21, 25, 29, 41, 42].

Consistent with the literature [25, 43], in the present study, *ompA* and *oma87* genes mediate the biosynthesis and integrity of the outer membrane of *P. multocida* were identified in all isolates (Figure-2c). The high prevalence of these genes demonstrates their importance to infection by *P. multocida* and survival in the host.

### Antimicrobial susceptibility of *P. multocida isolates*

Antimicrobials are still widely used to treat infections caused by *P. multocida*. However, today the problem of antibiotic resistance is becoming more and more serious not only in Vietnam [44], but throughout the world [45]. Bacterial cells can become resistant to antibiotics and drugs due to drug misuse in many aspects of life, such as antibiotic use in livestock, in disease treatment, and the use of unsuitable drugs, or drugs at the wrong dose. The antimicrobial susceptibility of *Pasteurella* species should be carefully monitored to detect resistance development. In this study, we found that the antibiotics enrofloxacin, ciprofloxacin, neomycin, and ofloxacin were the most effective in the treatment of *P. multocida* isolates as all strains were susceptible (100%) to these drugs (Table-3 and Figure-S1), followed by chloramphenicol (92.68%) and amikacin (90.24%). These results are in accordance with those obtained by Kumar *et al*. [46] and Sarangi *et al*. [21], who also found enrofloxacin, chloramphenicol and ofloxacin were the most effective against *P. multocida* isolated from sheep.

**Table-3:** The antimicrobial susceptibility profiles of *Pasteurella multocida* strains by disk diffusion test.

Antimicrobial agents	Sensitivity of isolates to antimicrobial agents		

	Sensitive (%)	Intermediate (%)	Resistant (%)
Amikacin	37 (90.24)	1 (2.44)	3 (7.32)
Amoxicillin	12 (29.27)	9 (21.95)	20 (48.78)
Ampicillin	11 (26.83)	8 (19.51)	22 (53.66)
Ciprofloxacin	41 (100)	0	0
Chloramphenicol	38 (92.68)	0	3 (7.32)
Erythromycin	8 (19.51)	6 (14.63)	27 (65.85)
Gentamicin	33 (80.48)	2 (4.88)	6 (14.63)
Kanamycin	35 (85.36)	1 (2.44)	5 (12.19)
Neomycin	41 (100)	0	0
Ofloxacin	41 (100)	0	0
Enrofloxacin	41 (100)	0	0
Tetracycline	12 (29.27)	8 (19.51)	21 (51.22)

*P. multocida=Pasteurella multocida*

**Figure-3:**
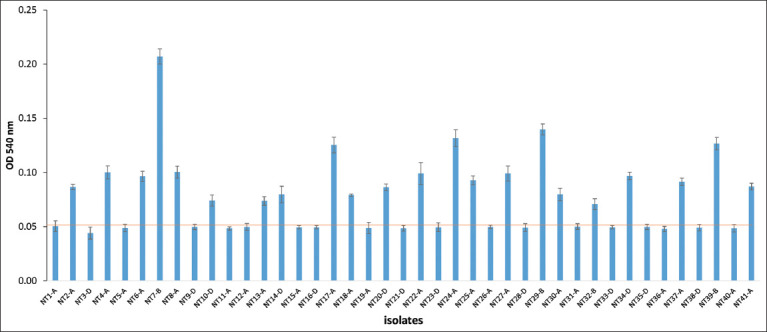
Biofilm formation of 41 *Pasteurella multocida* strains. The ability of isolates to form biofilm was measured at 540 nm using crystal violet after 24 h of growth in 96-well plates. Error bars indicate standard deviation from three independent experiments. The orange line indicates control measurement.

Conversely, the results shown in Table-3 indicate that erythromycin, tetracycline, and amoxicillin are less effective against *P. multocida* since the resistance rates are 65.85, 51.22, and 48.78%, respectively. On the other hand, Singh *et al*. [47] found the full susceptibility (100%) of amoxicillin and tetracycline against 12 *P. multocida* isolated from sheep. Similarly, Berge *et al*. [48] reported that all 19 *P. multocida* isolates were susceptible to amoxicillin. The difference may be due to grazing conditions in Vietnam, sheep are often raised with some other livestock and poultry such as cattle, chickens, pigs, and buffalo, which can lead to the transmission of resistant bacteria. Moreover, a recent report conducted on *P. multocida* isolated from pigs in Vietnam demonstrated higher resistance of isolates to amoxicillin and tetracycline (75.9 and 59%, respectively) compared to our results (48.78 and 51.22%, respectively) [5].

In our study, ampicillin was an inefficient antibiotic for treatment since 53.66% of *P. multocida* strains were resistant. This finding is consistent with that of Marru *et al*. [2], who found 53.1% of *P. multocida* strains were resistant to ampicillin and supported evidence from a previous Spanish study by Cid *et al*. [49], who reported that all 87 *P. multocida* strains isolated were resistant to ampicillin. In contrast, ampicillin has been reported as the most effective against *P. multocida* in other studies, of which all 28 *P. multocida* isolates were fully susceptible to ampicillin [17], of which ampicillin was significantly active against 177/186 *P. multocida* isolates [50].

Based on the antibiotic susceptibility test results (Table-3 and Figure-S1), 12.19 and 14.63% of isolates were resistant to kanamycin and gentamicin, respectively. Similar findings were also observed by Sarangi *et al*. [21] and the study of Singh *et al*. [47], who showed that more than 80% of *P. multocida* isolates were susceptible to these antibiotics. This finding is contrary to previous studies [46, 51] in which more than 60% of *P. multocida* isolates were resistant to kanamycin. Marru *et al*. [2] reported that all *Pasteurella* isolates were completely resistant to gentamicin. But the findings in the present study were very close to the result of Vu-Khac *et al*. [5], who found that the rate of resistance to gentamicin and kanamycin of porcine *Pasteurella* isolates in Vietnam were 14.5 and 15.7%, respectively.

Biofilm is a characteristic phenotype of many pathogenic bacteria; it helps them to survive in harsh environmental conditions and enhances their virulence and resistance to antibiotics [52]. Several pathogenic members of the *Pasteurellaceae*, including *P. multocida* have the ability to form biofilms [27, 53, 54] and such biofilms are often difficult to handle due to their limited antimicrobial activity [27]. In this study, as the results shown in Figure-3, 22 isolates (53.65%) were able to adhere to polystyrene and form biofilm, of which only 1 isolate (NT7) exhibited strong adherence, four isolates (NT17, NT24, NT29, and NT39) adhered moderately, and many of them, 18 isolates poorly adhered to polystyrene. On the other hand, 19 isolates (46.34%) could not produce biofilm at all. This finding is consistent with that of Emery *et al*. [53], who reported that 53.13% of *P. multocida* strains exhibited weak adherence to and 40.42% no adherence to polystyrene. However, the result was lower than those of Saha *et al*. [54], who found 81.82% of *P. multocida* strains could produce biofilm. Interestingly, the *tadD* gene is known to play an important role in adhesion and biofilm formation [34, 53], but in this study, there was no correlation between the prevalence of *tadD* and biofilm formation. 8/17 capsular A, 2/4 capsular B, and 1/4 capsular D isolates carrying *tadD* were able to form biofilm. Thus, the biofilm formation of *P. multocida* may be related to many complex genetic factors, in which the *tadD* and other genes may be tightly controlled by an unknown mechanism and need further research.

Interestingly, all four isolates carrying capsular B were able to produce biofilm, of which one formed weakly, one very strong production of biofilm and two formed biofilms moderately. These results support the findings of a great deal of the previous work in which capsular serotype group B of *P. multocida* isolates could form strong biofilms [55]. Prajapati *et al*. [55] also reported that 63% of serogroups A, and 100% of serogroups D of *P. multocida* isolates could not produce biofilm. This differs from the findings presented here: 58.33% (14/24) of *P. multocida* serotype A were able to form a biofilm, of which 8.33% (2/24) isolates could form moderate biofilms, and 50% (12/24) serotype A isolates were able to produce weak biofilms, whereas, 41.16% (10/24) of *P. multocida* serotype A isolates were unable to produce biofilm. Meanwhile, 30.76% (4/13) isolates carrying capsular D could form weak biofilms.

## Conclusion

This present study showed that *P. multocida* is frequently isolated from Phan Rang sheep in Vietnam and that isolates carry an abundance of virulence-associated genes. Several particular virulence genes, such as *toxA, pfhA, and tbpA* may be used as an epidemic marker for *P. multocida* and are also good candidates for vaccine development against *P. multocida*. Our findings also provide valuable information on the current susceptibility status and biofilm formation of *P. multocida* isolates.

## Authors’ Contributions

PVN and KCTN: Contributed to the conception of the study and drafted the manuscript. CTL, XHN and TMN: Contributed to sample collection, investigation, and data analysis. All authors have read and approved the final manuscript.

## Figures and Tables

**Figure-S1:**
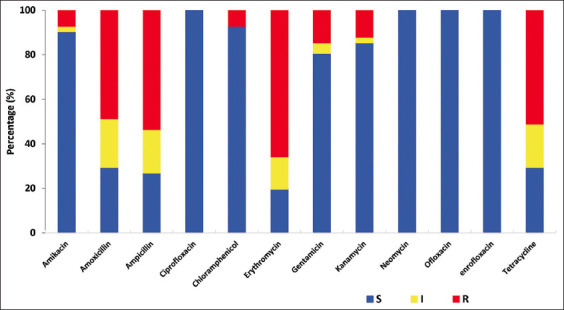
Antibiotic resistance profile of *Pasteurella multocida* isolates: Resistant (R) in red, intermediate (I) in yellow, and susceptible (S) in green color.
